# Disproportionate Elevated Troponin T Without Clinical Ischemia: A Diagnostic Challenge in the Emergency Department

**DOI:** 10.1155/carm/5452369

**Published:** 2026-02-25

**Authors:** Bryan Nicolalde, Ashraf Elamin, Dae Yong-Park, Michael DeCarolis, Samuel Hahn

**Affiliations:** ^1^ Department of Medicine, Robert Larner College of Medicine, University of Vermont, Burlington, Vermont, USA, uvm.edu; ^2^ Department of Medicine, Norwalk Hospital, Norwalk, Connecticut, USA, norwalkhospital.org; ^3^ Department of Medicine, Bridgeport Hospital/Yale University Program, Bridgeport, Connecticut, USA; ^4^ Department of Internal Medicine, Division of Cardiovascular Medicine, Yale School of Medicine, New Haven, Connecticut, USA, yale.edu; ^5^ Department of Emergency Medicine, Yale School of Medicine, New Haven, Connecticut, USA, yale.edu

## Abstract

**Background:**

Cardiac troponins (cTns) are essential for evaluating chest pain, but elevated levels can arise from noncardiac causes or laboratory artifacts. Confirmatory testing for assay interference is often unavailable for false‐positive cases, complicating diagnosis and management.

**Case Summary:**

A 62‐year‐old female presented with chest pain and disproportionally elevated high‐sensitivity troponin T (hs‐cTnT) levels (8396 ng/L). Clinical evaluation, including EKG, echocardiography, and nuclear myocardial perfusion images showed no evidence of acute ischemia. Noncardiac causes of elevation of troponins, as well laboratory artifacts, were considered as differential diagnostics. A clinically driven approach was adopted to resolve this case.

**Conclusion:**

In cases of disproportionally troponin elevation, a clinically driven approach incorporating risk stratification and alternative diagnostic tools, such as nuclear imaging, can effectively guide management and avoid unnecessary interventions.

## 1. Introduction

The measurement of cardiac biomarkers, particularly cardiac troponins (cTns), is a cornerstone of emergency department assessment for patients presenting with chest pain. Acute or chronic elevation of cTn isoforms I (cTnI) or T (cTnT) above the 99th percentile of the upper reference limit (URL) is a key criterion for defining myocardial injury [1]. Troponin elevation occurs due to both cardiac and noncardiac causes. While some practitioners refer to troponin elevations in noncardiac conditions as “false positives,” this terminology is inaccurate. Such elevations indicate genuine cardiac myocyte injury and subsequent troponin release secondary to a primary noncardiac disease [2].

The clinical context is crucial in interpreting elevated cTn levels, particularly when measurements appear disproportionately high in the absence of a consistent clinical correlation [3]. False‐positive elevations are caused by abnormal interactions between the troponin assay and blood components, or analyzer malfunction. Serum components’ mechanisms include interactions with fibrin clots, the formation of macrocomplexes of troponins with antibodies, interference with heterophile antibodies, elevation of alkaline phosphatase, hemolysis, rheumatoid factor, and myopathies [2, 3].

The International Federation of Clinical Chemistry Committee on Clinical Applications of Cardiac Biomarkers (IFCC C‐CBs) has proposed stepwise guidelines to address these laboratory artifacts [3]. However, implementing these guidelines in routine clinical practice is often challenging due to the need for confirmatory tests that require sending samples to specialized reference laboratories [3]. This process, although gold standard, can be time‐consuming, especially in emergency or urgent settings where rapid decision‐making is critical for patients presenting with chest pain.

In this case report, we describe a patient with markedly elevated troponin levels that were disproportionate to her clinical presentation. The confirmation of a false‐positive troponin elevation posed significant challenges due to limited laboratory capabilities. We applied a clinically driven approach to address this diagnostic challenge.

## 2. Case Description

A 62‐year‐old female with a history of insulin‐dependent Type 2 diabetes mellitus, hypertension, alcohol use disorder, chronic kidney disease stage IIIa, tobacco use disorder, and asthma presented to the emergency department for evaluation of chest pain. The pain was described as pressure‐like, nonradiating, and nonexertional, associated with shortness of breath and nausea but without diaphoresis. The episode lasted 1‐2 h and reported that the chest pain started following a stressful event. She denied palpitations, orthopnea, paroxysmal nocturnal dyspnea, or any viral prodrome. At the time of evaluation, she was free of chest pain. In the review of systems, she denied generalized weakness or myalgias. Her chart review revealed a previous nuclear cardiac stress test from 3 years before the presentation, which was normal.

Two months earlier, she was hospitalized for acute severe pancreatitis caused by GLP‐1 agonist use and alcohol use disorder. Her hospital course was complicated by oliguric renal failure due to acute tubular necrosis (ATN), requiring continuous renal replacement therapy (CRRT). Her kidney function recovered, and she was discharged with a creatinine of 1.8 mg/dL (baseline: 1.1 mg/dL). During that admission, her high‐sensitivity troponin T was 15 ng/L.

**FIGURE 1:**
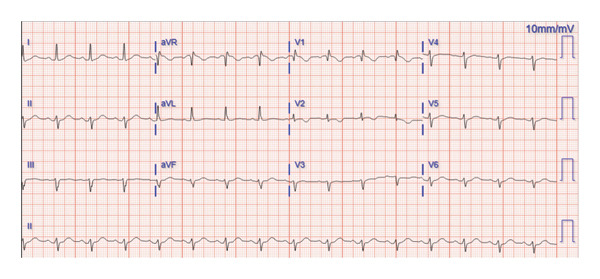
EKG on admission.

In the emergency department, her vital signs showed she was hemodynamically stable, with a blood pressure in the range of 90–100/50–70 mmHg and a heart rate of 80–90 bpm. She was afebrile and had Sp02 > 93% while breathing room air. Cardiac physical exam revealed no murmurs, heart rate regularly regular, no respiratory distress, no evidence of chest trauma, and no‐reproducible chest pain on palpation. Neurological and musculoskeletal examination was unremarkable. During the evaluation, there were no concerns of rhabdomyolysis. An initial electrocardiogram (EKG) revealed normal sinus rhythm and a chronic left anterior fascicular block without new ischemic changes (Figure 1). Our laboratory findings included a creatinine level of 1.1 mg/dL, an NT‐proBNP level of 258 pg/mL (decreased from a prior level of 615 pg/mL), and elevated high‐sensitivity troponin T of 8,396, 8,144, and 7,883 ng/L (ROCHE Elecsys Troponin T Gen 5) at first, second, and third sets, respectively. Total creatine kinase (CK) was initially 427 U/L and subsequently decreased to 288 U/L.

A transthoracic echocardiogram (TTE) revealed hyperdynamic left ventricular systolic function with an ejection fraction of 70% and no regional wall motion abnormalities. A CT pulmonary embolism protocol was obtained and resulted negative. The laboratory was consulted due to the inconsistency of clinical features and elevated troponins. Our laboratory was requested to perform measurements of troponin I, creatine kinase‐MB (CK‐MB), and polyethylene glycol (PEG) precipitation to evaluate for possible analytical interference, such as formation of macrotroponin complexes or interaction with heterophile antibodies. However, in our facility, those samples require external laboratory analysis, with an analysis time of several days, and the samples were ultimately not performed because the case was time‐sensitive.

Discussion with the laboratory did not reveal any abnormalities in quality control processes or any similar discordant results in other patients at that time, suggesting this was not a widespread assay issue.

**FIGURE 2:**
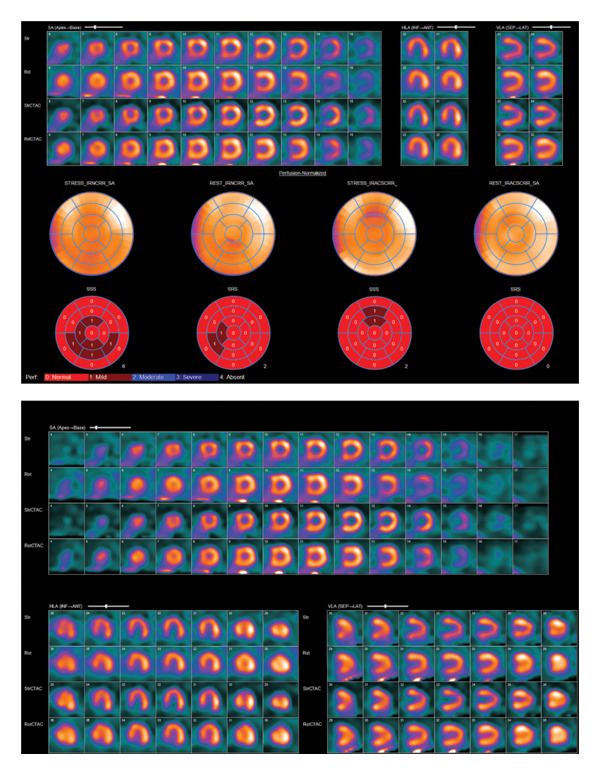
Nuclear SPECT myocardial perfusion test.

The patient was admitted for observation, and a nuclear cardiac stress test using regadenoson was performed (Figure 2). The study revealed normal SPECT myocardial perfusion imaging with no evidence of ischemia or infarction. Over a 72‐h observation period, she remained chest pain‐free and she was subsequently discharged.

## 3. Discussion

The evaluation of a patient with suspected ischemic chest pain in the emergency department, particularly those with cardiovascular high risk, most of the time is dependent of cardiac biomarkers such as high sensitive troponins. Troponins exist in three forms: troponin C, which binds to calcium; troponin I inhibits contraction; and troponin T, which binds the troponin complex to tropomyosin, facilitating contraction [4]. Troponin C is present in both cardiac and skeletal muscle, while troponins I and T are specific to the cardiac muscle. Troponins are present in the myocyte either bound to the sarcomere ∼95% or free in the cytosol ∼5% and leak into the circulation following myocardial injury [4, 5]. Once troponins are released in the bloodstream, the clearance depends upon liver reticulocyte mediated endocytosis and renal excretion [5]. Elevation of cTn may be seen in diverse cardiac conditions such as heart failure, pulmonary embolism, myocarditis, cardiomyopathies, arrhythmias, valvular heart disease, and cardiac contusions, as well as due to noncardiac causes, which include anemia, hypotension, hypoxia, noncardiac surgeries, and musculoskeletal myopathies. Elevation of troponins can also be secondary to delay in clearance such as secondary to acute kidney injury or in dialysis patients [2, 6]. The fourth universal definition of myocardial infarction has established a distinction between myocardial injury, characterized by elevated cardiac troponin levels above the 99th percentile, and myocardial infarction, which includes both myocardial injury and clinical signs of ischemia, new EKG changes, or imaging evidence of myocardial damage [1].

False elevation of troponins is sometimes attributed by physicians to noncardiac causes unrelated to acute ischemic events. However, true false‐positive results are rare and typically caused by laboratory artifacts. Cardiac troponins levels are measured using sandwich immunoassays, which involve a capture antibody that binds to one site of the cTn molecule and a detection antibody that binds to a different site. The detection signal is produced when both antibodies are attached to the same cTn molecule [5]. The IFCC C‐CB has identified various mechanisms responsible for false elevations of troponins. The most common cTn assay abnormality is when there is an outlier caused by fibrin clot and can be rule out with a successive analysis showing a different result [3]. When the false elevation persists the most common mechanism involves the presence of anticardiac troponin (anti‐cTn) antibodies in the serum, which can interfere with the assay process [3]. These antibodies are detected in 2%–20% of the individuals and are more frequently observed in patients with cardiac conditions [5]. Anti‐cTn antibodies may compete with assay antibodies for troponin binding, leading to assay signal interference [3]. Furthermore, these antibodies can promote the formation of immune complexes known as macrotroponins [3]. Due to the prolonged half‐life of immunoglobulins, macrotroponin complexes can persist in circulation much longer than free troponins. While free troponins typically have a plasma half‐life of approximately 120 min, macrotroponin complexes can remain detectable for days or even weeks [3, 5]. Although classically, the macrotroponins and heterophilic antibodies have been reported more with troponin I, in the literature, several cases involving troponin T are reported [5, 7–9].

Several methods have been proposed to detect interfering antibodies and improve the accuracy of troponin assay interpretation. However, these techniques are often manual, time‐consuming, and labor‐intensive and may delay critical time‐sensitive treatments. In some cases, they require sending samples to specialized reference laboratories, resulting in waiting periods ranging from hours to days for analysis and results. Proposed methods include precipitating large molecules using polyethylene glycol, removing IgG with protein A/G spin columns, blocking cross‐linking antibodies with heterophile blocking reagents, and separating molecules based on molecular weight through gel filtration chromatography or sucrose gradient ultracentrifugation [3]. On the other hand, interpreting false results could end in performing unnecessary invasive procedures or administration of medications.

This case highlights the diagnostic challenge of interpreting markedly elevated troponin levels in the emergency setting when there is a lack of consistent clinical correlation. Recognizing the disproportionate elevation in troponin levels, the clinical team promptly engaged the laboratory for further evaluation and repeat analysis, which yielded similar results. Additional testing, including measurements of troponin I and CK‐MB, as well as precipitation testing with PEG, was requested; however, these tests were unavailable on‐site and required processing at an external laboratory, resulting in delays which affected patients’ disposition in the emergency department and inpatient setting. This scenario highlights the importance of close collaboration between clinicians and laboratory specialists in addressing suspected analytical interferences. Laboratory review did not identify concurrent quality control issues or similar discordant troponin results on other patients at that moment. While confirmatory tests are invaluable for identifying false‐positive results, the practical limitations of laboratory resources, especially in urgent clinical settings, necessitate a predominantly clinical approach to decision‐making. In such situations, the integration of laboratory findings with clinical context and noninvasive diagnostic tools plays a pivotal role in guiding patient management and avoiding unnecessary interventions.

In this case, several factors guided our decision‐making process. At the time of evaluation, the patient’s chest pain had resolved, the EKG was unremarkable for ischemic changes, and a STAT echocardiogram demonstrated no wall motion abnormalities. As a result, despite elevated troponin levels, the clinical presentation did not meet the criteria for myocardial infarction, which would have necessitated immediate cardiac catheterization and/or anticoagulation. Additionally, a review of the patient’s medical history revealed a negative cardiac nuclear stress test performed 3 years prior. The extent of troponin elevation provided important diagnostic insight. While elevations greater than 1000–10,000 ng/L are typically indicative of large myocardial infarction, myocarditis, or stress cardiomyopathy, these conditions are often accompanied by abnormal findings on EKG or echocardiography and a compatible clinical context [10]. It is also noteworthy that echocardiography has a reported sensitivity of approximately 90% and specificity of 80% in detecting wall motion abnormalities in acute coronary syndromes [11]. Given the elevated cardiac biomarkers and the unresolved etiology of chest pain, the patient was admitted for observation. We utilized CK testing as it was the only alternative biomarker immediately available, which, although elevated, was not proportional to the marked elevation in high‐sensitivity troponin levels. This disparity further supported the decision to exclude myocardial infarction as the cause of troponin elevation. In addition, despite myopathies could cause elevation of CK and cTnT, our patient lacks clinical symptoms/signs of myopathies such as rash, muscular pain, and generalized or focal weakness. Also, the elevation of cTnT in myopathies has been reported to be lower than that in our case, with reported values up to 44.5 ng/L in Elecsys assays. Also, in such cases, CK has been in higher concentrations around 1000 U/L [6, 12]. Other etiologies such us pulmonary embolism were ruled out with the CT pulmonary embolism protocol and the patient lack of criteria for sepsis.

During the patient’s evaluation, we opted for a nuclear medicine test due to a negative EKG, echo results, and inconclusive troponin levels. The utility of the rest phase of radionuclide myocardial perfusion imaging (rMPI) has been well‐validated in assessing patients presenting with acute chest pain and nondiagnostic EKG or echo findings. rMPI with technetium‐99m (Tc‐99m) sestamibi or Tc‐99m tetrofosmin involves radiolabeled tracers that accumulate in the myocardium in proportion to myocardial blood flow. When these agents are administered during or shortly after the resolution of chest pain, ischemic myocardial regions demonstrate reduced radiotracer uptake, reflected by decreased radioactive counts [13–15]. In these scenarios, it has been seen that sensitivity is 93% with a negative predictive value of 99% [16]. In addition, the stress phase provided us valuable information regarding the presence of significant coronary obstruction with a sensitivity and specificity in between 80% and 90% [17].

In our case, the diagnosis of analytical interference was presumptive, based on marked discordance between extreme troponin elevation and the absence of clinical, electrocardiographic, and imaging evidence of myocardial injury. We determined that most of the increase in troponin levels was due to analytical issues after ruling out other potential causes. Additionally, some of the elevation may be related to noncardiac factors, such as a delay in clearing troponins due to recent episode of acute on chronic kidney injury and possibly from skeletal muscle regeneration.

The absence of definitive laboratory confirmation represents a limitation of this report. However, this case illustrates the real‐world challenges faced by many hospitals where specialized testing is not immediately available and highlights how thoughtful clinical reasoning can support diagnostic decision‐making in the presence of discordant biomarkers.

## 4. Conclusion

In our case, the inability to confirm the etiology of falsely elevated troponins due to laboratory limitations highlights a common clinical scenario where gold‐standard confirmation may not be practical. By adopting a clinically driven approach, we relied on patient risk stratification and complementary diagnostic modalities, including EKG, echocardiography, and nuclear medicine testing, to evaluate and rule out acute ischemia. This approach highlights the importance of integrating multiple diagnostic tools in the absence of definitive laboratory results.

## Funding

No funding was received for this manuscript.

## Consent

The patient has given her consent for submission and publication of this case report including figures and associated text in accordance with the Committee on Publications Ethics (COPE) guidelines.

## Conflicts of Interest

The authors declare no conflicts of interest.

## Data Availability

Deidentified data related to this case report are available from the corresponding author upon reasonable request. Requests will be reviewed by the corresponding author to ensure they comply with patient confidentiality agreements and ethical guidelines.
